# Predicting prognosis for adults with depression using individual symptom data: a comparison of modelling approaches

**DOI:** 10.1017/S0033291721001616

**Published:** 2023-01

**Authors:** J. E. J. Buckman, Z. D. Cohen, C. O'Driscoll, E. I. Fried, R. Saunders, G. Ambler, R. J. DeRubeis, S. Gilbody, S. D. Hollon, T. Kendrick, E. Watkins, T.C. Eley, A. J. Peel, C. Rayner, D. Kessler, N. Wiles, G. Lewis, S. Pilling

**Affiliations:** 1 Research Department of Clinical, Educational & Health Psychology, Centre for Outcomes Research and Effectiveness (CORE), University College London, 1-19 Torrington Place, London, UK; 2 iCope – Camden & Islington Psychological Therapies Services – Camden & Islington NHS Foundation Trust, St Pancras Hospital, London, UK; 3 Department of Psychiatry, University of California, Los Angeles, Los Angeles, CA, USA; 4 Department of Clinical Psychology, Leiden University, Leiden, The Netherlands; 5 Statistical Science, University College London, 1-19 Torrington Place, London, UK; 6 Department of Psychology, School of Arts and Sciences, 425 S. University Avenue, Philadelphia PA, USA; 7 Department of Health Sciences, University of York, Seebohm Rowntree Building, Heslington, York, UK; 8 Department of Psychology, Vanderbilt University, Nashville, TN, USA; 9 Primary Care, Population Sciences and Medical Education, Faculty of Medicine, University of Southampton, Aldermoor Health Centre, Southampton, UK; 10 Department of Psychology, University of Exeter, Sir Henry Wellcome Building for Mood Disorders Research, Perry Road, Exeter, UK; 11 Social, Genetic and Developmental Psychiatry Centre, Institute of Psychiatry, Psychology & Neuroscience, King's College London, London, UK; 12 Centre for Academic Primary Care, Population Health Sciences, Bristol Medical School, University of Bristol, Canynge Hall, Bristol, UK; 13 Centre for Academic Mental Health, Population Health Sciences, Bristol Medical School, University of Bristol, Oakfield House, Bristol, UK; 14 Division of Psychiatry, University College London, Maple House, London, UK; 15 Camden & Islington NHS Foundation Trust, St Pancras Hospital, London, UK

**Keywords:** Depressive symptoms, major depression, network analysis, prediction modelling, prognosis

## Abstract

**Background:**

This study aimed to develop, validate and compare the performance of models predicting post-treatment outcomes for depressed adults based on pre-treatment data.

**Methods:**

Individual patient data from all six eligible randomised controlled trials were used to develop (*k* = 3, *n* = 1722) and test (*k* = 3, *n* = 918) nine models. Predictors included depressive and anxiety symptoms, social support, life events and alcohol use. Weighted sum scores were developed using coefficient weights derived from network centrality statistics (models 1–3) and factor loadings from a confirmatory factor analysis (model 4). Unweighted sum score models were tested using elastic net regularised (ENR) and ordinary least squares (OLS) regression (models 5 and 6). Individual items were then included in ENR and OLS (models 7 and 8). All models were compared to one another and to a null model (mean post-baseline Beck Depression Inventory Second Edition (BDI-II) score in the training data: model 9). Primary outcome: BDI-II scores at 3–4 months.

**Results:**

Models 1–7 all outperformed the null model and model 8. Model performance was very similar across models 1–6, meaning that differential weights applied to the baseline sum scores had little impact.

**Conclusions:**

Any of the modelling techniques (models 1–7) could be used to inform prognostic predictions for depressed adults with differences in the proportions of patients reaching remission based on the predicted severity of depressive symptoms post-treatment. However, the majority of variance in prognosis remained unexplained. It may be necessary to include a broader range of biopsychosocial variables to better adjudicate between competing models, and to derive models with greater clinical utility for treatment-seeking adults with depression.

## Introduction

Depression affects ~320 million people worldwide every year (Thornicroft et al., 2017; Vos et al., 2016). Despite the existence of effective treatments, roughly half of depressed patients do not recover with the first treatment they are given. This can lead to disengagement and poor long-term prognoses (Buckman et al., 2018; Judd et al., 1998). Providing accurate predictions about the likelihood of treatment response for patients would be of great value, informing clinical management and giving patients and clinicians desired information (Hayden, Windt Van Der, Cartwright, Côté, & Bombardier, 2013; Morgan, Reavley, & Jorm, 2014). However, there are a lack of accurate, validated prognostic models for adults in treatment for depression (Cohen & DeRubeis, 2018). Central to this vacancy in the literature are methodological inconsistencies, debates about how best to develop predictive models, and what variables to include in such models. Recently, the field has begun to reach consensus on how to best test the utility of predictive models, for example, by evaluating them in datasets that are separate from those used for model development (Adibi, Sadatsafavi, & Ioannidis, 2020; Dwyer, Falkai, & Koutsouleris, 2018; Harrell, Lee, & Mark, 2004; Moons et al., 2015; Steyerberg et al., 2010).

One factor consistently found to be associated with prognosis of depression is the severity of depressive symptoms pre-treatment (Bower et al., 2013; Driessen, Cuijpers, Hollon, & Dekker, 2010; Fournier, Derubeis, Hollon, Shelton, & Fawcett, 2010; Weitz et al., 2015). This is often captured with sum scores on depressive symptom scales. However, depression is heterogeneous (Fried & Nesse, 2015a) so utilising symptom level data might provide more nuanced information on patients experiences of depression, and consequently improve the accuracy of prognostic predictions (Boschloo, van Borkulo, Borsboom, & Schoevers, 2016; Fava, Ruini, & Belaise, 2007; Fried & Nesse, 2014, 2015b). Network theory (Borsboom & Cramer, 2013; Fried & Cramer, 2017) has given rise to an approach that can capture the relationships between individual symptoms. These relationships could reflect potential causal pathways, thereby elucidating maintenance mechanisms that could be targeted with treatment, and might therefore inform prognosis (Borsboom, 2017). The arrangement and inter-relationships of symptoms within networks have most often been captured with one or more measures of centrality – i.e. the interconnectedness of each symptom with other symptoms in the network (Bringmann et al., 2019; Fried, Epskamp, Nesse, Tuerlinckx, & Borsboom, 2016).

A recent study used centrality metrics to weight individual items of a depressive symptom questionnaire, which when summed together created a new, or weighted, sum score. A regression model using this weighted sum score was found to outperform a model containing the original sum score in an exploratory analysis (Boschloo et al., 2016). Other studies have utilised centrality metrics to predict changes in particular symptoms over time (Boschloo et al., 2016; Koenders et al., 2015; van Borkulo et al., 2015; Wichers & Groot, 2016), or predict post-treatment outcomes (Berlim, Richard-Devantoy, Dos Santos, & Turecki, 2020; Elliott, Jones, & Schmidt, 2020). However, such studies have not tested the developed models against simpler comparative models, nor have they tested the predictive utility of the models in completely external data (Dwyer et al., 2018; Harrell et al., 2004; Webb et al., 2020), or adhered to recent conventions for the transparency of conducting such research by following pre-registered analysis plans or protocols (Collins, Reitsma, Altman, & Moons, 2015). Therefore, the extent to which the use of centrality metrics can add incremental value in prognostic models remains unclear. The present paper aims to fill this gap and further the consideration of the development of models that can be translated into clinical settings.

There are several potentially equally valid ways to estimate item centrality in network models. We will therefore investigate several methods that have been used in the recent network modelling literature. One method uses the estimated arrangement of items into communities of highly partially correlated items, we will compare this to a model in which it is assumed that there is a single latent factor. We will use these methods to investigate the benefit of using item centrality scores and factor loadings to create weighted sum scores, and compare these to an unweighted regression model, and to a penalised regression model, as these are typical methods used to develop predictive models. We will then compare all of these methods against models that use all the individual items rather than sum scores, and to a simple null model (Boschloo et al., 2016). In this way, this study aims to develop, validate and compare the predictive performance of prognostic models for depressed adults in primary care, based on pre-treatment data including individual symptoms of depression.

## Methods

The methods for the present study were pre-registered (https://osf.io/vzk65/). We have reported the details in accordance with TRIPOD, brief details are given below, and further information including a TRIPOD checklist is available in the online Supplementary materials.

### Ethical considerations and trial registrations

All included studies were granted ethical approvals and all participants gave informed consent (online Supplementary Table S5). No additional NHS ethical approval was required for this study: HRA reference 712/86/32/81.

### Participants

The dataset for this study comes from a larger project investigating prognosis for adults with depression in primary care, the project involved systematic literature searches to form an individual patient dataset (IPD) from eligible randomised controlled trials (RCTs; Buckman et al., 2020). The final searches were conducted on 1 December 2020 (Buckman et al., 2021b). Studies were included if they were RCTs that recruited adults with depression in primary care, and used the Revised Clinical Interview Schedule (CIS-R) (Lewis, Pelosi, Araya, & Dunn, 1992) to collect depressive and anxiety symptom data and determine diagnoses. This was to ensure uniformity across the studies in the items available for the predictive models. From our previous work we found that the CIS-R is the most commonly used comprehensive measure of this kind in studies of depression in primary care (Buckman et al., 2021a). Studies also had to use the Beck Depression Inventory Second Edition (BDI-II) (Beck, Steer, & Brown, 1996) to collect individual symptoms of depression. Six RCTs met inclusion criteria and were split such that half (*k* = 3, *n* = 1722) would form a dataset to develop the predictive models (the ‘training set’) and half (*k* = 3, *n* = 1136, of which 918 had outcome data and were used to evaluate the models as detailed below) would form a separate dataset to test the models (the ‘test set’). See online Supplementary Table S1 and Supplementary Fig. S1, for details of each study. It was decided that studies with similar types of treatments would be split across the training and test sets (with all data from one study going into the training set and all data from the other study going into the test set), and where this was the case, those with the larger sample sizes would go into the training data.

### Predictors and measures

Predictors varied depending on the model used, as detailed below (Table 1). Models either included total scores (with items either weighted or unweighted) or individual items from the BDI-II. All models used total scores for the eight anxiety subscales from CIS-R (generalised anxiety, worry, compulsions, obsessions, phobic anxiety, health anxiety, somatic concerns, and panic; with items either weighted or unweighted), and total scores for alcohol use, social support and life events. In previous studies using similar data it has been found that these factors are independently associated with poorer prognoses, and may have utility in predicting treatment outcomes (Buckman et al., 2021a; Buckman et al., 2021b; O'Driscoll et al., 2021). The total scores for the social support, life events and alcohol measures were required instead of the individual items. There was strong topological overlap between the social support items, and all eight items were highly correlated with one another, which would have led to inflated centrality scores were the individual items included in the network models. Further, the level of multi-collinearity went beyond pairs of items, so instead of removing those leading to high multi-collinearity, it was necessary to use the sum score as the best measure of this construct. Modelling binary items into a network is possible but not when using the fused graphical least absolute shrinkage and selection operator (LASSO) (FGL) method adopted here to deal with between-study heterogeneity, so the total score from the life events scale was used. As alcohol misuse was an exclusion criterion for some of the eligible RCTs, there was near zero variability for many of the items. The sum score therefore represented the best measure of alcohol use.

**Table 1.** Description of the modelling approaches for the primary outcome

Type of approach	Weighting approach	Model number	Method	Predictors included	Description
Weighted sum scores	One-step EI (FGL)	1	OLS	CIS-R weighted sum score for anxiety subscales, BDI-II weighted score, SSS score, LE score and AUDIT-PC score	Sum of all edges connected to the focal node used to weight items to construct weighted sum scores
	Two-step EI (FGL)	2	OLS	CIS-R weighted sum score for anxiety subscales, BDI-II weighted score, SSS score, LE score and AUDIT-PC score	Sum of all edges connected to either the focal node or any other node directly connected to the focal node
	PC/PR (FGL)	3	OLS	CIS-R weighted sum score for anxiety subscales, BDI-II weighted score, SSS score, LE score and AUDIT-PC score	the geometric mean between the participation coefficient (PC) and participation ratio (PR)
	CFA	4	OLS	CIS-R weighted sum score for anxiety subscales, BDI-II weighted score, SSS score, LE score and AUDIT-PC score	Factor loadings from CFA were used as weights to develop the weighted total scores
Unweighted sum scores	Shrinkage	5	ENR	CIS-R unweighted sum score for anxiety subscales, BDI-II score, SSS score, LE score and AUDIT-PC score	ENR built using the unweighted total scores
	None	6	OLS	CIS-R unweighted sum score for anxiety subscales, BDI-II score, SSS score, LE score and AUDIT-PC score	OLS model with unweighted total scores on the baseline measures
Individual symptoms	Shrinkage	7	ENR	CIS-R anxiety subscale items, BDI-II individual items, SSS score, LE score and AUDIT-PC score	ENR model using all of the individual items of BDI-II, anxiety sub-scores of CIS-R and total scores of other measures
	None	8	OLS	CIS-R anxiety subscale items, BDI-II individual items, SSS score, LE score and AUDIT-PC score	OLS regression model with items assessing the same symptoms included in weighted models.
Null model	None	9	OLS	Mean BDI-II sum score	Mean BDI-II score in training set studies used as prediction for all cases in test set

BDI-II, Beck Depression Inventory Second Edition; CFA, confirmatory factor analysis; CIS-R, Revised Clinical Interview Schedule; EI, expected influence; ENR, elastic net regularised regression; FGL, fused graphical LASSO; LE, life events; OLS, ordinary least squares; PC/PR, geometric mean between the participation ratio and participation coefficient; SSS, social support scale.

The null models used the BDI-II total score only. See online Supplementary Table S2 for details of the measures.

### Outcomes

The primary outcome was the BDI-II score at 3–4 months post-baseline. The secondary outcome was remission at 3–4 months post-baseline, defined as a score of ⩽10 on the BDI-II. In all but one of the six studies, assessors and analysts were blind to treatment allocation when collecting these data.

### Data analysis

Missing data were imputed in the training set for all variables with <30% missing, using the ‘missForest’ package in R (Stekhoven & Bühlmann, 2012). In the test set, the same approach was used but outcome data were not imputed. The maximum amount of missing data of any of the variables used in the predictive models here, at baseline in any of the six studies was 0.83%. In the test set, 218 participants were missing outcome data and were excluded from the analyses. For one study whose data were included in the training set, ‘COBALT’, BDI-II was not collected at 3–4 months. These scores were imputed using the methods above based on all available variables in that study including baseline BDI-II scores and patient health questionnaire-9 (PHQ-9) scores, 3-month PHQ-9 scores, 6-month BDI-II and PHQ-9 scores, and 12-month BDI-II and PHQ-9 scores (see online Supplementary for additional details).

### Model building

Nine models were constructed in the training set (Table 1) for both primary and secondary outcomes, so 18 models were fitted overall.

For the first four models, we developed separate weighted sum scores for the CIS-R anxiety subscales by summing together coefficient weights for each of the eight subscales, and for the BDI-II by summing together coefficient weights for each of the 21 BDI-II items. Weighted sum scores for the CIS-R anxiety subscales and BDI-II, and coefficient weights for the total scores for social support, life events, and alcohol were used as predictors by entering them into regression models (ordinary least squares (OLS) for the primary outcome and logistic regression for the secondary outcome). This follows a method used by others to develop predictive models from networks (Boschloo et al., 2016). As described below, models 5 and 7 were based on a method that develops model weights internally (elastic net regularised regression (ENR)). Models 6 and 8 used the original, unweighted scores as a means of comparison. Model 9 was a null model, detailed further below.

### Network analyses

There are two established ways to estimate a network model across several datasets. First, pool the data and estimate a model. Second, a recent innovation in network methods, the FGL (Costantini & Epskamp, 2017b; Fried et al., 2018), which estimates a model on several datasets and obtains one network. The FGL uses extended Bayesian information criterion, LASSO regularised regression models run separately for each study, and the models are then fused together to get a single network penalising differences among corresponding edge weights in the study networks. It is therefore considered better suited to deal with between-study heterogeneity (Costantini et al., 2019), and so was the method used here. For further details on how to estimate and interpret network structures and a comprehensive review of the network literature (see Epskamp & Fried, 2018; Robinaugh, Hoekstra, Toner, & Borsboom, 2020). For models 1–3, the FGL model was estimated using item-level data from CIS-R anxiety subscales and the BDI-II with tuning parameters selected through 10-fold cross validation (Costantini & Epskamp, 2017a; Danaher, Wang, & Witten, 2014). Centrality metrics derived from the FGL were used to construct weights after re-scaling these to be between 0 and 1. The three methods for determining coefficient weights from the estimated networks were: model (1) one-step expected influence (EI: sum of all edges connected to the focal node); model (2) two-step EI (sum of all edges connected to either the focal node or any other node directly connected to the focal node) (Robinaugh, Millner, & McNally, 2016); and model (3) the geometric mean of the participation coefficient (PC) and participation ratio (PR) (Letina, Blanken, Deserno, & Borsboom, 2019). See online Supplementary materials for details. The EI metrics are widely used and have recently been proposed to be informative for predicting treatment outcomes (Berlim et al., 2020; Elliott et al., 2020). PC/PR is a newer approach, which is thought to be more sensitive to the use of different scale measures within the same network as it takes the community structure (multidimensionality) into account (Letina et al., 2019). This is important here as we used measures of severity beyond depressive symptoms, given their importance for prognosis (Buckman et al., 2021a; Lorenzo-Luaces, Rodriguez-Quintana, & Bailey, 2020).

### Confirmatory factor analyses

Model 4 was a unidimensional confirmatory factor analytic (CFA) model that assumes the data come from a single dimensional latent construct (in contrast to model 3, which is based on a Walktrap algorithm that identifies densely connected communities of items via random walks). Factor loadings were rescaled to be between 0 and 1 and summed to develop the weighted total scores.

### Penalised regression analyses

Model 5 was an ENR model built using the unweighted total scores on the same scales that were used for models 1–4. In ENR, variables are selected and model weights are assigned through the use of LASSO and ridge penalisations. Parameter space was searched using 10-fold cross-validation to identify the optimal settings for these parameters before building the final model (Friedman, Hastie, & Tibshirani, 2010; Webb et al., 2020). Model 7 was an ENR using all of the individual items from the BDI-II and the CIS-R anxiety subscales, and total scores for life events, social support and alcohol use.

### Non-penalised regression analyses

Two simple comparison models were constructed using non-penalised regression (OLS regression for continuous outcomes and logistic regression for binary outcomes). Model 6 used the unweighted total scores on the five baseline measures, and model 8 used the same items as model 7.

### Null models

A null model was built for each outcome for the purpose of comparison. For the primary outcome, this used the mean 3–4-month BDI-II score in the training set as the prediction for all patients in the test set, and for the secondary outcome the proportion of participants in remission in the training set was used as the prediction for all patients in the test set.

### Sensitivity analyses

In order to assess the impact of having to impute the 3–4-month BDI-II outcomes for the COBALT study, we conducted two sensitivity analyses. All analyses using BDI-II as the outcome were re-done excluding COBALT from the training dataset. Then, a different way of capturing depressive symptoms at 3–4 months was calculated based on a method of converting scores from different depressive symptom measures to a single comparable score; the PROMIS T-score (Choi, Schalet, Cook, & Cella, 2014), using a multidimensional item-response theory-based conversion tool (Fischer & Rose, 2016), see online Supplementary for further details.

### Model evaluation

Models were first evaluated in the full test set comprising three studies (TREAD, IPCRESS and MIR), and then separately in each of the three study samples. They were also evaluated in a 10-fold internal cross-validation of the full training set data.

For the continuous outcomes, there were three metrics used to evaluate the models: the amount of variance explained (*R*^2^), the root mean-squared error (RMSE), and the mean absolute error (MAE). For the binary outcome, there were two metrics used to evaluate the models: the area under the receiver operating characteristic curve, and Brier scores. Since the *R*^2^ in this study is a comparison of the predicted BDI-II score values to the mean BDI-II score at 3–4 months in the test set, and the training and test set BDI-II score means at 3–4 months differed, it was expected that some models might have *R*^2^ values less than zero. There are limits to the inferences that can be drawn from the above metrics due to the variability in the modelling schemes that were applied (e.g. in which variables were made available; the number of variables made available; whether or not network analysis or factor analysis was used to create weighted sum scores; and whether or not penalised regression was applied to the variables that were made available). To make these performance metrics more accessible, we have provided three visualisations that demonstrate the potential clinical relevance of each model. For each of the eight models (excluding the null model) the predicted BDI-II scores at 3–4 months were arrayed from the lowest to the highest, then: (1) we plotted the observed BDI-II score at 3–4 months against the predicted score in groups (‘bins’) of *n* = 50; (2) predicted scores were split into categories of severity in line with delineations made by the originators of the scale (Beck et al., 1996) (i.e. scores between 0 and 13 were considered minimal, 14 and 19 mild, 20 and 28 moderate, and 29 and 63 severe), and the rate of remission observed in the test set samples was calculated for each category; and (3) to provide a more granular visualisation of remission we plotted the observed percentage of participants in remission against BDI-II predicted scores at 3–4 months, again in bins of *n* = 50.

## Results

### Characteristics of the included studies

Six RCTs met inclusion criteria, three formed the training dataset (*n* = 1772) and three formed the test dataset (*n* = 1136, of which *n* = 918 had outcome data available for analyses), see online Supplementary Fig. S1 for flow of studies and online Supplementary Table S1 for details of each study.

### Descriptive statistics

Descriptive statistics and comparisons of the distributions of socio-demographics and markers of severity across the training set and test set samples are provided in Table 2. There were some differences between the training and test datasets: fewer people of non-White ethnicities were in the test set, and more of the training sample were unemployed. On average the test set participants had more comorbid disorders although a higher proportion of the training set sample had comorbid panic disorder, specific phobias, or chronic fatigue syndrome. The mean score on the AUDIT-PC was higher in the test set. In addition, the mean BDI-II scores were higher in the test set (by 2.47 points at baseline and 3.53 points at 3–4 months). This corresponded with a large difference in the proportions of each sample reaching remission: 48.83% in the training set and 32.53% in the test set.

**Table 2.** Descriptive statistics for training and test set samples, and comparison of the two datasets

		Train set	Test set	*t*-test or χ^2^
Self-reported baseline characteristics	Factor	*N* (%) or mean (s.d.)	*N* (%) or mean (s.d.)	*p* value
	*Sample size*	*1772*	*1136*	
Age in years	Mean (s.d.)	42.1 (14.0)	43.2 (14.3)	0.051
Gender	Female	1131 (65.7)	769 (67.8)	0.237
	Male	59 (34.3)	365 (32.2)	
Ethnicity	White	1613 (93.7)	1085 (95.6)	0.028
	Non-White	109 (6.33)	50 (4.41)	
Employment status	Employed	996 (57.8)	643 (56.7)	0.002
	Not seeking employment	379 (22.0)	306 (27.0)	
	Unemployed	347 (20.2)	185 (16.3)	
Marital status	Married/cohabiting	819 (47.6)	560 (49.3)	0.608
	Single	560 (32.5)	351 (30.9)	
	No longer married	343 (19.9)	225 (19.8)	
Number of recent life events	Mean (s.d.)	1.39 (1.26)	1.28 (1.20)	0.021
Social support total	Median (interquartile range)	21 (18 to 24)	22 (18 to 24)	0.752
AUDIT-PC score	Mean (s.d.)	2.57 (2.87)	3.13 (3.26)	<0.001
Past antidepressant use	No	537 (31.2)	371 (32.7)	0.408
	Yes	1185 (68.8)	765 (67.3)	
CIS-R Sum of anxiety Subscales score	Mean (s.d.)	13.7 (6.85)	13.9 (6.31)	0.437
CIS-R durations	Depression	3.38 (1.44)	3.48 (1.25)	0.056
	Average anxiety duration	2.14 (1.00)	2.13 (0.97)	0.780
Comorbid anxiety disorders	Mean (s.d.)	2.03 (1.17)	2.19 (1.05)	0.0002
Agoraphobia	No	1554 (89.7)	991 (87.3)	0.052
	Yes	178 (10.3)	144 (12.7)	
Chronic fatigue syndrome	No	615 (35.7)	348 (30.6)	0.005
	Yes	1107 (64.3)	788 (69.4)	
Generalised anxiety disorder	No	701 (40.7)	492 (43.4)	0.162
	Yes	1021 (59.3)	643 (56.7)	
Mixed anxiety and depressive disorder	No	1241 (72.1)	798 (70.3)	0.292
	Yes	481 (27.9)	338 (29.8)	
Obsessive compulsive disorder	No	1477 (85.8)	962 (84.7)	0.42
	Yes	245 (14.2)	174 (15.3)	
Panic disorder	No	1562 (90.7)	1061 (93.4)	0.01
	Yes	160 (9.3)	75 (6.60)	
Specific phobias	No	1406 (81.7)	967 (85.1)	0.015
	Yes	316 (18.4)	169 (14.9)	
Baseline BDI-II score	Mean (s.d.)	29.5 (11.1)	31.9 (9.45)	<0.001
Three–four months BDI-II score	Mean (s.d.)	14.4 (11.4)	17.9 (12.4)	<0.001
Remission 3–4 months	No	742 (51.2)	621 (67.7)	<0.001
	Yes	708 (48.8)	297 (32.4)	
Baseline PROMIS score	Mean (s.d.)	70.3 (8.38)	73.3 (6.36)	<0.001
Three–four months PROMIS score	Mean (s.d.)	60.1 (11.5)	60.4 (12.5)	0.499

### Formation of the models

The weights given to the individual items for models 1–4 are shown in online Supplementary Table S6. Final model coefficients are presented in online Supplementary Tables S7 and S8.

### Comparison of model performance

After the models were developed they were evaluated using the test dataset. Despite slight differences in the formation of some of the models, they made very similar predictions of who would get better (remit) and by what magnitude (BDI-II score) at 3–4 months. To illustrate this, the predictions produced for the primary outcome by the models were highly correlated (all correlation coefficients above *r* = 0.90 for models 1–6 and above *r* = 0.75 for models 7–8) see online Supplementary Fig. S2.

**Fig. 1.**
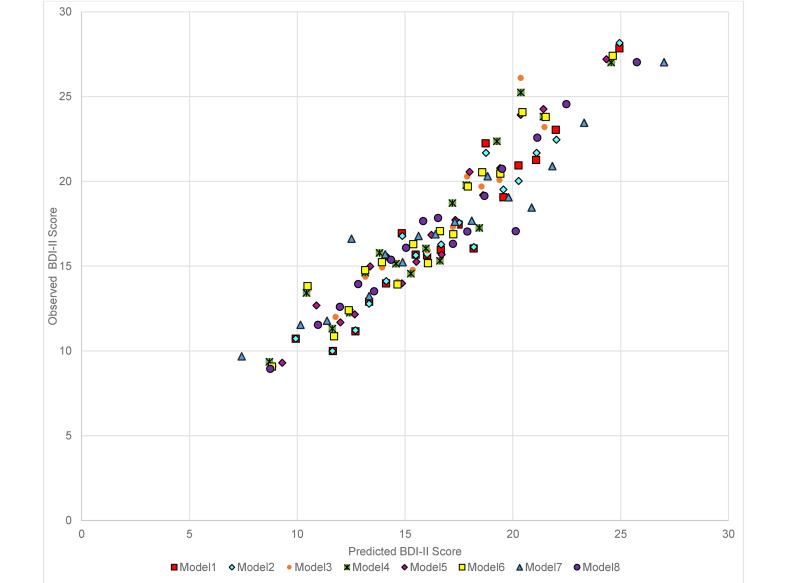
Predicted and observed BDI-II score at 3–4 months in combined test set data (*n* = 918) by the eight models (excluding the null model) built in the Training set data.

For the primary outcome (BDI-II score at 3–4 months post-baseline) in the combined test sets, the RMSE was similar for models 1–6 (the largest difference was between model 2 which had the lowest RMSE and model 4, =0.057) with a slightly higher RMSE for the OLS individual-item model (model 8) (difference between model 2 and model 8 = 0.214). Models 1–8 made similar predictions for those with BDI-II scores at 3–4 months that were <18 or >25, but diverged more in the predictions for those with scores between 18 and 24, see Fig. 1 (for ease of presentation, results are displayed for groups of 50 participants, each point shows the mean predicted and observed score for the 50 participants closest to that point on the graph). All models (1–8) had lower RMSE scores than the null model (ranging from 0.944 for the difference between models 8 and 9 to 1.158 for the difference between models 2 and 9), see Table 3. The amount of variance explained by models 1–7 was again very similar with *R*^2^ values between 0.157 and 0.169. Model 8 (*R*^2^ = 0.109) explained less variance, but all models had *R*^2^ values well above the null model (*R*^2^ = −0.01). MAE values were similar for models 1–7 (ranging between 9.089 for model 5 and 9.173 for model 7). MAE was slightly higher in model 8 (=9.279) and higher again in the null model (9.935), see Table 3. For the secondary outcome there was a similar pattern to the results, although the null model (9) had a similar Brier score to models 1–7 and this was slightly lower than that of model 8 (=0.246), see online Supplementary Table S3. There were greater variations between the models in the separate test set studies than in the overall test set and for all models (1–9). Additionally, the RMSE and MAE scores were lower, and *R*^2^s were higher, in the internal cross-validation than in the external test set data.

**Table 3.** Performance of the models predicting BDI-II scores at 3–4 months post-baseline in the test datasets individually and combined

		All studies combined (*n* = 918)			IPCRESS (*n* = 206)			MIR (*n* = 424)			TREAD (*n* = 288)			Internal cross-validation		
Type of approach	Model	RMSE	*R*^2^	MAE	RMSE	*R*^2^	MAE	RMSE	*R*^2^	MAE	RMSE	*R*^2^	MAE	RMSE	*R*^2^	MAE
Weighted sum scores	1. EI 1-step	11.285	0.168	9.122	11.642	0.171	9.572	11.216	0.174	8.993	11.127	0.137	8.990	9.995	0.216	7.991
	2. EI 2-Step	11.281	0.169	9.119	11.646	0.170	9.575	11.209	0.175	8.987	11.122	0.137	8.985	9.992	0.216	7.989
	3. PR/PC	11.326	0.162	9.097	11.626	0.173	9.526	11.226	0.177	9.053	11.253	0.117	8.856	9.941	0.223	7.940
	4. CFA	11.338	0.160	9.100	11.655	0.169	9.548	11.219	0.175	9.041	11.284	0.112	8.865	9.953	0.221	7.946
Unweighted sum scores	5. ENR^a^	11.311	0.165	9.089	11.638	0.171	9.541	11.232	0.173	9.046	11.189	0.127	8.827	9.946	0.223	7.950
	6. OLS	11.319	0.163	9.091	11.631	0.172	9.544	11.220	0.175	9.045	11.237	0.119	8.836	9.947	0.222	7.944
Individual symptoms	7. ENR^b^	11.359	0.157	9.173	11.869	0.138	9.798	11.201	0.178	9.075	11.216	0.123	8.871	9.881	0.233	7.886
	8. OLS	11.495	0.137	9.279	12.192	0.090	10.084	11.225	0.174	9.094	11.375	0.098	8.976	9.904	0.230	7.881
Null	9. Null	12.439	−0.010	9.935	12.852	−0.011	10.396	12.544	−0.031	9.993	11.975	0.000	9.521	11.270	−0.001	9.026

CFA, confirmatory factor analysis; EI, expected influence; ENR, elastic net regularised regression; MAE, mean absolute error; OLS, ordinary least squares; PC, participation coefficient; PR, participation ratio; RMSE, root mean-squared error.

Note there is no calculation of *r^2^* for the null model as all there was no variability in prediction.

a Parameters were set at (*ᾳ* = 0.82 and *λ* = 0.20).

b Parameters were set at (*ᾳ* = 0.05 and *λ* = 2.0).

**Fig. 2.**
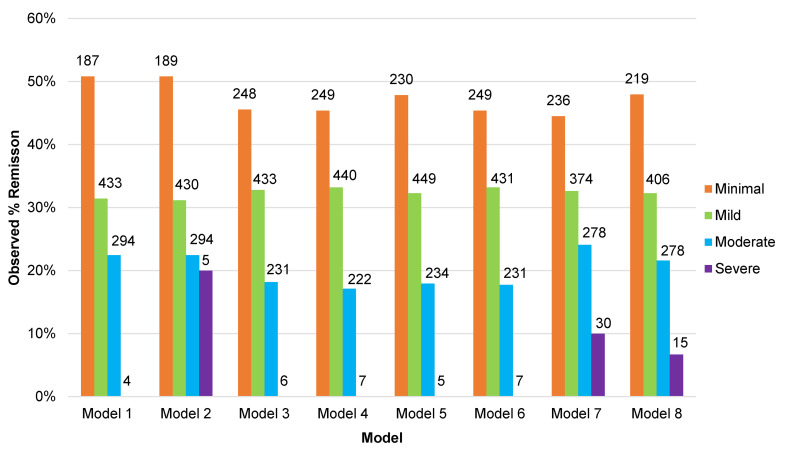
Proportion of participants in remission at 3–4 months post-baseline in the test set studies (*n* = 918) by predicted category of depressive severity at 3–4 months, for each of the eight models.

In order to evaluate the potential clinical relevance of the models we determined the observed proportion of participants in remission at 3–4 months based on the predicted score made by each model (online Supplementary Fig. S3), and the same based on categories of severity of symptoms taken from the predicted scores (see Fig. 2). From these figures we can see that when the models predicted high BDI-II scores at 3–4 months the chances of being in remission were very low. Models 7 and 8 predicted more participants would have severe depression at 3–4 months than the other models. When the models predicted minimal symptoms (BDI-II scores <10) the observed rate of remission was around 50%. There were few differences between the models overall, although greater variations in the observed rates of remission between the models for patients predicted to have mild to moderate BDI-II scores at 3–4 months.

Sensitivity analyses did not lead to any substantive differences in our findings, see online Supplementary Tables S2 and S3.

## Discussion

There were few differences in the performance of the majority of the predictive models. The first seven models all outperformed the null models on all metrics for primary and secondary outcomes. Those using weighted or unweighted sum scores (the first six models) performed better in the held-out test data than the individual-item models did, particularly model 8 (the OLS regression model using all of the individual BDI-II score items and eight CIS-R anxiety subscale scores instead of the sum scores for each). Any of the eight models could be used to predict the severity of depressive symptoms at 3–4 months after starting treatment based on pre-treatment data. The large difference in observed remission rates between those predicted to have high compared to low BDI-II scores at 3–4 months informs the potential clinical relevance of these models.

### Strengths and limitations

This study was the first to provide robust tests of the ability of centrality statistics from FGL networks and factor loadings from a factor analytic model to develop weighted total scale scores to inform predictive models of treatment outcomes. This is something that has been proposed as a promising method for using individual symptom data to build informative predictive models (Boschloo et al., 2016). We tested these methods against *bone fide* predictive models and simple comparison models, and in entirely held-out (test) data, and found there to be little evidence of any advantage to the above approaches. We used a large individual patient data dataset comprising six RCTs with a variety of widely available treatments for depression, all of the RCTs were situated in primary care, and five were pragmatic trials, increasing the generalisability of these results (Rothwell, 2005). However, the variability in the samples between the studies may have limited the overall performance of the models. We included a range of psychopathology measures at baseline, not just depression symptoms from a single measure, as there is good evidence that such factors are associated with prognosis for depressed adults (Buckman et al., 2021a; Buckman et al., 2021b). We also used the most commonly utilised comprehensive measure of depressive and anxiety symptoms and diagnoses from RCTs of depression in primary care, to minimise bias in harmonising data, and ensure a broad range of depressive and anxiety based symptoms could be included in the models we developed.

However, there were a number of limitations. Not all important covariates were controlled for: we did not include data on durations of depression or anxiety despite their associations with prognosis for adults with depression (Buckman et al., 2021a; Lorenzo-Luaces et al., 2020). Including such data would have led to problems of multi-collinearity with the symptoms of the individual comorbid anxiety disorders experienced by each participant, and across durations of anxiety disorders and depression, biasing centrality estimates and factor loadings for models 1–4. The intercepts and coefficient weights provided in the online Supplementary materials could be used to derive prognostic predictions for future depressed patients using models developed here. However, there were large amounts of variance in the outcome that could not be explained by any of the models. This is consistent with other studies that developed and validated predictive models for patients with depression (Delgadillo, Huey, Bennett, & McMillan, 2017; Webb et al., 2020). Some of the unexplained variance is likely due to measurement error and other factors, including those that better capture the biopsychosocial complexity of depression. We speculate that such factors would need to be included before the predictive models could more accurately predict prognosis for any individual patient (Fried & Robinaugh, 2020). Crucially, for this study, such improvements in accuracy may also have been required for us to find substantial differences in the performance of the modelling schemes.

In this study, predictions of prognosis were made regardless of the type of treatment given, as this may have most utility at the point when patients are seeking treatment, i.e. before a decision on the type of treatment has been made (Buckman et al., 2021a; Marwood, Wise, Perkins, & Cleare, 2018). Although the train and test set studies were split such that where possible, there was a balance of treatment types across the datasets, it may be the case that the models would perform differently between types of treatments. Future studies might address differential model performance by treatment type but adequate data to do so were not available here (Fisher, Carpenter, Morris, Freeman, & Tierney, 2017b).

The present study used prognostic outcomes including depressive symptom severity at 3–4 months and remission, but both of these relied on sum scores from the BDI-II. As the BDI-II items or sum score were used in the development of the predictive models it might have been informative to consider model performance with an entirely separate but clinically meaningful outcome such as functioning, quality of life, or mental pain (Fava et al., 2019); data on such outcomes were not available here. In addition, models here used IPD but the networks were estimated based on aggregated data, a number of studies have shown the potential utility of using idiographic networks to predict outcomes for individual patients (Fisher & Boswell, 2016; Fisher, Medaglia, & Jeronimus, 2018; Fisher, Reeves, Lawyer, Medaglia, & Rubel, 2017a), this may yet prove the most fruitful avenue for using networks to inform prognostic models which are able to outperform classic regression models of the same factors.

## Implications and conclusions

Prognoses generated by the models developed here could be informative for depressed patients seeking treatment in primary care. However, there were few differences between the models, with no clear advantage in using individual items over sum scores, or in using network models or factor analytic models to weight individual items, in order to derive prognostic predictions. This may represent a limitation of the available data, or of the modelling approaches (that e.g. rely on estimating linear relations). In all of the models, the degree of inaccuracy in their predictions might be unacceptable to any individual patient. There were clear differences in the number of people reaching remission when the models predicted patients would have particularly low or high scores, but the models performed less well with BDI-II scores between 18 and 25. It may be informative for future studies to test the utility in giving more intensive treatments or more regular clinical reviews for patients with these mid-range scores, particularly if there is uncertainty about the value of doing so based on clinical severity. It is noteworthy that all of the models utilised both depressive and anxiety symptom data, and all but one included the total score from the life events scale, and six of the eight included the social support scale score. It might therefore be informative for prognosis to assess for these factors routinely in clinic. The individual-item models outperformed the others in the internal cross-validation data suggesting that narrow constructs (e.g. anhedonia) might be more informative for prognosis than broad constructs (e.g. depression), but issues of measurement error arise, particularly with the validity of the single items to measure each narrow construct. The findings presented here also highlight the importance of external validation in accounting for issues of overfitting.

## Supplementary material

For supplementary material accompanying this paper visit https://doi.org/10.1017/S0033291721001616. click here to view supplementary material
