# Supplementary material for: Predicting prognosis for adults with depression using individual symptom data: a comparison of modelling approaches
Source: Psychol Med. 2021 May 6;53(2):408–18. doi: 10.1017/S0033291721001616 (PMC9899563; doi:10.1017/S0033291721001616)

## Supplementary Materials

### Further details of data analyses

#### Pre-processing

Due to overlap between symptoms measured by the CIS-R and BDI-II, pre-processing will identify multicollinearity. Items to be removed will be chosen based on having lower variability in the pair. Additionally, assumptions relating to near zero variance, approximately equal variance of nodes, asymmetrical distributions will be assessed. Items will removed if they clearly violate assumptions across 2 or more training studies.

The pre-processing will apply to the three network informed weighted models and the unidimensional confirmatory factor analytic (CFA) model, any item removed from the network at the pre-processing stage will be weighted to zero.

For the ENR, ‘dummy coded’ variables will be created for each category of any non-continuous/ordinal variables.

#### Potential Deviations from Pre-processing Procedure

If we find that the pre-processing stages above related to removing variables due to multi-collinearity or assumption violations for the network modelling result in numerous variables being removed from one set of models (the network models) but not from others, we will consider using less-conservative means of pre-processing so as to not invalidate model comparisons. For example, we might suggest that only those variables that violate network-modelling assumptions in all of the training set studies be removed rather than those that do so in just two of the studies.

#### Missing Data

Missing data will be imputed using the “missForest” package (D. J. Stekhoven & Bühlmann, 2012) in R Studio (R Core Team, 2013). This uses a random forest model to impute missing data on all types of variables (continuous, categorical and binary) generating a single dataset with imputed values taken by averaging across a large number of regression trees. The imputation model will be run separately in each of the six RCTs, the results from which will be merged to form the two datasets (train and test). For studies in the train-set, all individuals with ≥30% missingness on baseline variables and all variables with ≥30% missingness across all participants will be excluded. Missing baseline data and outcomes for the remaining cases will be imputed. The primary analyses in the test-set studies will be completers only (those without outcomes will be excluded) although the systematically missing BDI-II scores at 3-4 months post-baseline in COBALT will be interpolated using “missForest” using data at baseline, 6-8 months and the PHQ-9 scores at 3-4 months. As with the training data, cases in the test sample with ≥30% missingness on baseline variables will be excluded and imputation will be performed via random forest. However, outcome data will not be used to inform imputation of missing baseline data for the primary analyses in the test sample.

#### Consistency Checks and Additional Model Evaluation

In addition to considering the performance of the models using the metrics specified in the ‘Model Evaluation’ section above the predictions of outcome for each model were compared in a correlation matrix, the weights applied to the predictor variables were also compared.

#### Software & Packages

Data handling and cleaning prior to the development of the Dep-GP database was performed in Stata 15.0 (StataCorp LP., 2017). All data pre-processing, imputation and analyses for the outlined study will be performed in R (R Core Team, 2019).

The R packages to be used are:

- Bootnet (Epskamp, Borsboom, & Fried, 2018)
- Caret (Kuhn et al., 2016)
- EGAnet (Golino & Epskamp, 2017)
- EstimateGroupNetwork (Costantini & Epskamp, 2017)
- glmnet (Friedman, Hastie, Simon, & Tibshirani, 2018)
- missForest (D. J. Stekhoven & Bühlmann, 2012)
- mgm (Haslbeck & Waldorp, 2019)
- networktools (Jones, 2018)
- qgraph (Epskamp, Costantini, Haslbeck, Cramer, & Borsboom, 2019)
- mirt (Chalmers, 2012)
- lavaan (Rosseel, 2012)

**Supplementary Table 1.** Description of included studies

| **Study** | **Sample and Recruitment** | **Interventions (N)** | **Outcome Measure (N for analysis)** | **Dataset** |
| --- | --- | --- | --- | --- |
| COBALT (Wiles et al., 2013) | Adults aged 18-75 with treatment resistant depression, scoring ≥14 BDI-II, recruited between November 2008 and September 2010 from 73 general practices in urban and rural settings in three UK centres: Bristol, Exeter, and Glasgow | TAU (n=235) vs CBT+TAU (n=234) | BDI-II also PHQ-9 (n=469) | Train |
| GENPOD (Glyn Lewis et al., 2011) | Adults aged 18-74 with depressive episode, recruited by GPs in three UK centres: Bristol, Birmingham and Newcastle between October 2005 and February 2008. | Citalopram (n=298) vs Reboxetine (n=303) | BDI-II (n=601) | Train |
| PANDA (Gemma Lewis et al., 2019) | Adults presenting with low mood or depression to GP in last 2 years, free of ADM for 8 weeks up to baseline. Recruited between January 2015 and August 2018 from 179 primary care surgeries in four UK cities (Bristol, Liverpool, London, and York) | Sertraline (n=323) vs Placebo (n=329) | PHQ-9 also BDI-II (n=652 | Train |
|  |  |  |  |  |
| TREAD (Chalder et al., 2012) | Adults aged 18-69 who met diagnostic criteria for MDD and scored ≥14 on BDI-II. Recruited from 65 primary care centres in Bristol and Exeter, UK, from August 2007 to October 2009. | TAU (n=179) vs Physical Activity + TAU (n=182) | BDI-II (n=288) | Test |
| IPCRESS (D. Kessler et al., 2009) | Adults scoring ≥14 BDI-II and GP confirmed diagnosis of depression. Recruited from 55 general practices in Bristol, London, and Warwickshire, between October 2005 and February 2008 | iCBT (n=148) vs TAU (n=147) | BDI-II (n=206) | Test |
| MIR (D. S. Kessler et al., 2018) | Adults ≥18 taking SSRIs or SNRIs at adequate dose for≥ 6 weeks, and scored ≥14 on BDI-II. Recruited from general practices surrounding four centres in Bristol, Exeter, Hull, and Keele/North Staffordshire, UK, between August 2013 and October 2015. | Mirtazapine (n=241) vs Placebo (n=239) | BDI-II also PHQ-9 (n=424) | Test |

Abbreviations: ADM – antidepressant medication; BDI-II – Beck Depression Inventory; GP – General Practitioner; iCBT (internet based therapist delivered cognitive behavioural therapy); MDD – Major Depressive Disorder; PHQ-9 – Patient Health Questionnaire 9-item version; SNRI – Serotonin-Norepinephrine Reuptake Inhibitor; SSRI – Selective Serotonin Reuptake Inhibitor; TAU – treatment as usual

**Supplementary Table 2.** Measures used across the studies of the Dep-GP IPD database

| **Measure** | **Details** | **Scores and Cut-offs for Remission** |
| --- | --- | --- |
| The CIS-R (Glyn Lewis et al., 1992) | Consists of 14 symptom subsections scored 0-4, five of which measure depressive symptoms: core features of depression, depressive thoughts (scored 0-5), fatigue, concentration/forgetfulness, and sleep. Nine sections measure anxiety symptoms: generalized anxiety, worry, irritability, obsessions, compulsions, health anxiety, somatic concerns, phobic anxiety (split into agoraphobia, social phobia, and specific phobia), and panic. A final section measures general health, impairment and weight change. Here only eight anxiety subscales were used, irritability was not used given the similarity between this and the agitation item of the BDI-II. | The total score ranges from 0-57 with a cut-off of ≥12 used to indicate likely common mental disorder, primary and secondary diagnoses using ICD-10 criteria are given as are binary indictors of diagnosis for all the disorders assessed. Scores of <12 among those that were previously depressed can be used to indicate remission. |
| Beck Depression Inventory 2^nd^ Edition (BDI-II) (Beck et al., 1996) | Consists of 21 items to assess depressive symptoms, each item is scored 0-3. | There is a maximum score obtainable of 63, and a cut-off of ≥10 is used indicate significant symptoms of depression, scores of <10 are therefore used to indicate remission in those that were previously depressed/scored ≥10. |
| Patient Health Questionnaire 9-item version (PHQ-9) (Kroenke, Spitzer, & Williams, 2001) | This is a depression screening measure, with respondents asked to rate how often they have been bothered by each of the nine symptom items over the preceding two weeks. Each item is scored 0-3 | There is a maximum score of 27 with a cut-off of ≥10 is used to indicate “caseness” for depression, a score of 9 or below for those that were previously depressed is therefore considered to indicate remission |
| Social Support Scale - adapted by authors of RCTs (D. Kessler et al., 2009) included in this IPD by adding one item to the Health and Lifestyles Survey Social Support Measure (Cox et al., 1987) | An 8-item instrument (the first seven of which are from the Health and Lifestyles Survey) assessing the degree to which participants rated the social support of their friends and family in each of the following domains: 1) being accepted for who one is; 2) feeling cared about; 3) feeling loved; 4) feeling important to them; 5) being able to rely on them; 6) feeling well supported and encouraged by them; 7) being made to feel happy by them; and 8) feeling able to talk to them whenever one might like. Items are scored 1-3, with total scores ranging from 8-24; higher scores indicate higher levels of perceived social support. The authors of the Health and Lifestyles Survey suggested the maximum score for social support (which was 21 on that scale) indicated ‘no lack of social support’, scores between 18-20 indicated a ‘moderate lack of social support’, and scores of 17 or below indicated a ‘severe lack of social support’. | N/A |
| Life events: adapted by the authors of the Adult Psychiatric Morbidity Surveys (McManus, Bebbington, Jenkins, & Brugha, 2016) based on the Social Readjustment Rating Scale (Holmes & Rahe, 1967) | Participants are asked to respond yes/no to whether they have suffered any of eight events within the last six months e.g. a death/bereavement; being physically attacked/injured; or going through a divorce/separation. Each item is scored yes (1) or no (0) and the total score is the sum of all the items. | N/A |
| Alcohol use: the alcohol use disorder identification test primary care version (AUDIT-PC) (Piccinelli et al., 1997). | Used to assess alcohol misuse, this includes five items scored 0-4. A cut-off of ≥5 indicates hazardous alcohol use that may be harmful to one’s health | N/A |
| All measures apart from the PHQ-9 were used in all six studies, PHQ-9 was used in three studies (COBALT, MIR, & PANDA), here it was only used for imputation and in the formation of the PROMIS T-Score in sensitivity analyses. | | |

**Supplementary Table 3.** Performance of the models predicting remission at 3-4 months post-baseline in the test datasets individually and combined.

|  |  | **All studies combined (n=918)** | | **IPCRESS (n=206)** | | **MIR (n=424)** | | **TREAD (n=288)** | | Internal Cross-validation | |
| --- | --- | --- | --- | --- | --- | --- | --- | --- | --- | --- | --- |
| Type of approach | Model | AUC | Brier | AUC | Brier | AUC | Brier | AUC | Brier | AUC | Brier |
| Weighted Sum-scores | 1. EI 1-step | 0.628 | 0.227 | 0.670 | 0.211 | 0.645 | 0.228 | 0.591 | 0.237 | 0.731 | 0.208 |
|  | 2. EI 2-Step | 0.628 | 0.227 | 0.669 | 0.211 | 0.645 | 0.228 | 0.591 | 0.237 | 0.731 | 0.208 |
|  | 3. PR/PC | 0.633 | 0.234 | 0.681 | 0.209 | 0.649 | 0.230 | 0.593 | 0.259 | 0.737 | 0.206 |
|  | 4. CFA | 0.631 | 0.237 | 0.672 | 0.211 | 0.652 | 0.229 | 0.588 | 0.267 | 0.735 | 0.207 |
| Unweighted Sum-scores | 5. ENR* | 0.626 | 0.234 | 0.653 | 0.235 | 0.648 | 0.232 | 0.592 | 0.237 | 0.724 | 0.243 |
|  | 6. Logistic Regression | 0.632 | 0.236 | 0.675 | 0.210 | 0.647 | 0.229 | 0.593 | 0.264 | 0.737 | 0.206 |
| Individual Symptoms | 7. ENR⸷ | 0.618 | 0.233 | 0.668 | 0.233 | 0.625 | 0.232 | 0.590 | 0.236 | 0.716 | 0.242 |
|  | 8. OLS | 0.599 | 0.246 | 0.642 | 0.219 | 0.608 | 0.236 | 0.585 | 0.278 | 0.738 | 0.207 |
| Null | 9. Null | N/A | 0.237 | N/A | 0.239 | N/A | 0.235 | N/A | 0.239 | N/A | 0.237 |

Abbreviations: AUC – Area Under the receiver operating characteristic Curve; CFA - Confirmatory Factor Analysis; EI - Expected Influence; ENR - Elastic Net Regularized Regression; OLS - Ordinary Least Squares; PC - Participation Coefficient; PR -Participation Ratio; *parameters were set at (ᾳ = 0.05 and λ = 2.0); ⸷ parameters were set at (ᾳ = 0.05 and λ = 2.0)

**Supplementary Table 4.** Performance of the models predicting PROMIS T-score scores at 3-4 months post-baseline in the test datasets individually and combined.

|  |  | **Test set (n with complete data at 3-4 months post-baseline)** | | | | | | | | | | | | |  |  |  |
| --- | --- | --- | --- | --- | --- | --- | --- | --- | --- | --- | --- | --- | --- | --- | --- | --- | --- |
|  |  | **All studies combined (n=918)** | | | **IPCRESS (n=206)** | | | **MIR (n=424)** | | | **TREAD (n=288)** | | | Internal Cross-validation | | | |
| Type of approach | **Model** | RMSE | R^2^ | MAE | RMSE | R^2^ | MAE | RMSE | R^2^ | MAE | RMSE | R^2^ | MAE | RMSE | | R^2^ | MAE |
| Weighted Sum-scores | 1. EI 1-step | 11.855 | 0.103 | 9.332 | 13.624 | 0.039 | 10.644 | 11.077 | 0.143 | 8.920 | 11.514 | 0.081 | 8.940 | 10.169 | | 0.152 | 7.999 |
|  | 2. EI 2-Step | 11.853 | 0.104 | 9.329 | 13.628 | 0.039 | 10.647 | 11.072 | 0.143 | 8.914 | 11.509 | 0.081 | 8.937 | 10.167 | | 0.152 | 7.998 |
|  | 3. Geometric-mean PR/PC | 11.843 | 0.105 | 9.447 | 13.575 | 0.046 | 10.593 | 11.081 | 0.142 | 8.999 | 11.563 | 0.081 | 9.327 | 10.117 | | 0.161 | 7.961 |
|  | 4. CFA | 11.863 | 0.102 | 9.477 | 13.613 | 0.041 | 10.639 | 11.067 | 0.144 | 8.978 | 11.563 | 0.073 | 9.327 | 10.122 | | 0.160 | 7.978 |
| Unweighted Sum-scores | 5. ENR* | 11.859 | 0.103 | 9.473 | 13.599 | 0.043 | 10.635 | 11.085 | 0.141 | 8.991 | 11.539 | 0.077 | 9.299 | 10.114 | | 0.161 | 7.961 |
|  | 6. OLS | 11.851 | 0.104 | 9.462 | 13.599 | 0.043 | 10.625 | 11.076 | 0.143 | 8.980 | 11.524 | 0.079 | 9.287 | 10.115 | | 0.161 | 7.961 |
| Individual Symptoms | 7. ENR⸷ | 12.082 | 0.069 | 9.538 | 14.379 | -0.070 | 10.949 | 11.128 | 0.135 | 8.939 | 11.503 | 0.082 | 9.348 | 10.025 | | 0.176 | 7.869 |
|  | 8. OLS | 12.232 | 0.046 | 9.643 | 14.706 | -0.120 | 11.107 | 11.185 | 0.126 | 8.969 | 11.622 | 0.063 | 9.525 | 10.035 | | 0.174 | 7.867 |
| Null | 9. Null | 12.522 | 0.000 | 10.007 | 13.995 | -0.014 | 11.360 | 12.022 | 0.000 | 9.719 | 12.045 | -0.006 | 9.399 | 11.045 | | 0.000 | 8.775 |

Abbreviations: CFA - Confirmatory Factor Analysis; EI - Expected Influence; ENR - Elastic Net Regularized Regression; MAE - Mean Absolute Error; OLS - Ordinary Least Squares; PC - Participation Coefficient; PR -Participation Ratio; RMSE - Root Mean-Squared Error. Note there is no calculation of r^2^ for the Null model as all there was no variability in prediction. *parameters were set at (ᾳ = 0.0 and λ = 0.40); ⸷ parameters were set at (ᾳ = 0.0 and λ = 2.0)

**Supplementary Table 5.** Performance of the models predicting BDI-II scores at 3-4 months post-baseline in the test datasets individually and combined. Models were only developed in two training set studies, excluding COBALT.

|  |  | **Test set (n with complete data at 3-4 months post-baseline)** | | | | | | | | | | | | |  |  |  |
| --- | --- | --- | --- | --- | --- | --- | --- | --- | --- | --- | --- | --- | --- | --- | --- | --- | --- |
|  |  | **All studies combined (n=918)** | | | **IPCRESS (n=206)** | | | **MIR (n=424)** | | | **TREAD (n=288)** | | | Internal Cross-validation | | | |
| Type of approach | **Model** | RMSE | R^2^ | MAE | RMSE | R^2^ | MAE | RMSE | R^2^ | MAE | RMSE | R^2^ | MAE | RMSE | | R^2^ | MAE |
| Weighted Sum-scores | 1. EI 1-step | 11.441 | 0.145 | 9.173 | 11.603 | 0.176 | 9.424 | 11.578 | 0.121 | 9.241 | 11.115 | 0.138 | 8.895 | 9.906 | | 0.191 | 7.869 |
|  | 2. EI 2-Step | 11.435 | 0.146 | 9.170 | 11.607 | 0.176 | 9.427 | 11.569 | 0.123 | 9.234 | 11.111 | 0.139 | 8.891 | 9.903 | | 0.191 | 7.868 |
|  | 3. Geometric-mean PR/PC | 11.550 | 0.129 | 9.202 | 11.615 | 0.174 | 9.442 | 11.542 | 0.127 | 9.270 | 11.515 | 0.075 | 8.930 | 9.848 | | 0.200 | 7.832 |
|  | 4. CFA | 11.566 | 0.126 | 9.217 | 11.604 | 0.176 | 9.404 | 11.619 | 0.115 | 9.313 | 11.458 | 0.084 | 8.944 | 9.848 | | 0.200 | 7.817 |
| Unweighted Sum-scores | 5. ENR* | 11.595 | 0.126 | 9.225 | 11.625 | 0.173 | 9.424 | 11.613 | 0.116 | 9.320 | 11.462 | 0.084 | 8.944 | 9.849 | | 0.200 | 7.821 |
|  | 6. OLS | 11.540 | 0.130 | 9.207 | 11.587 | 0.178 | 9.392 | 11.596 | 0.119 | 9.307 | 11.423 | 0.090 | 8.926 | 9.850 | | 0.200 | 7.818 |
| Individual Symptoms | 7. ENR⸷ | 11.552 | 0.129 | 9.262 | 11.896 | 0.134 | 9.782 | 11.463 | 0.139 | 9.229 | 11.431 | 0.089 | 8.939 | 9.865 | | 0.198 | 7.836 |
|  | 8. OLS | 11.683 | 0.109 | 9.373 | 12.186 | 0.091 | 10.047 | 11.489 | 0.135 | 9.256 | 11.597 | 0.062 | 9.064 | 9.899 | | 0.192 | 7.834 |
| Null | 9. Null | 12.664 | -0.047 | 9.918 | 13.076 | -0.046 | 10.414 | 12.871 | -0.086 | 10.081 | 12.044 | -0.012 | 9.322 | 11.021 | | -0.001 | 8.740 |

Abbreviations: CFA - Confirmatory Factor Analysis; EI - Expected Influence; ENR - Elastic Net Regularized Regression; MAE - Mean Absolute Error; OLS - Ordinary Least Squares; PC - Participation Coefficient; PR -Participation Ratio; RMSE - Root Mean-Squared Error. Note there is no calculation of r^2^ for the Null model as all there was no variability in prediction. *parameters were set at (ᾳ = 0.0 and λ = 0.45); ⸷ parameters were set at (ᾳ = 0.0 and λ = 2.0)

| **Variable** | **Model** | | | |
| --- | --- | --- | --- | --- |
|  | **EI 1-Step (1)** | **EI 2-Step (2)** | **PC_PR (3)** | **CFA (4)** |
| Anxiety (cisr) | 0.773 | 0.757 | 0.724 | 0.165 |
| Compulsions (cisr) | 0.638 | 0.626 | 0.716 | 0.172 |
| Health anxiety (cisr) | 0.656 | 0.620 | 0.812 | 0.173 |
| Obsessions (cisr) | 0.547 | 0.543 | 0.598 | 0.181 |
| Panic (cisr) | 0.895 | 0.874 | 0.891 | 0.188 |
| Phobia (cisr) | 0.810 | 0.803 | 0.797 | 0.168 |
| Somatic (cisr) | 0.439 | 0.437 | 0.602 | 0.186 |
| Worry (cisr) | 0.719 | 0.704 | 0.696 | 0.176 |
| Sadness (BDI) | 0.955 | 0.943 | 0.978 | 0.182 |
| Pessimism (BDI) | 0.736 | 0.792 | 0.801 | 0.181 |
| Failure (BDI) | 0.814 | 0.872 | 0.718 | 0.172 |
| Loss of pleasure (BDI) | 0.785 | 0.834 | 0.844 | 0.180 |
| Guilt (BDI) | 0.861 | 0.881 | 0.851 | 0.194 |
| Punishment (BDI) | 0.813 | 0.825 | 0.860 | 0.176 |
| Self dislike (BDI) | 0.696 | 0.732 | 0.515 | 0.182 |
| Self criticism (BDI) | 0.853 | 0.866 | 0.763 | 0.156 |
| Suicidal thoughts (BDI) | 0.741 | 0.750 | 0.811 | 0.173 |
| Crying (BDI) | 0.578 | 0.586 | 0.733 | 0.163 |
| Agitation (BDI) | 0.698 | 0.686 | 0.709 | 0.184 |
| Loss of Interest (BDI) | 0.800 | 0.820 | 0.857 | 0.184 |
| Indecisiveness (BDI) | 0.736 | 0.765 | 0.828 | 0.186 |
| Worthlessness (BDI) | 1.000 | 1.000 | 0.736 | 0.169 |
| Loss of energy (BDI) | 0.900 | 0.916 | 0.888 | 0.156 |
| Sleep (BDI) | 0.492 | 0.486 | 0.547 | 0.177 |
| Irritability (BDI) | 0.825 | 0.817 | 0.830 | 0.177 |
| Concentration (BDI) | 0.912 | 0.921 | 0.823 | 0.170 |
| Fatigue (BDI) | 0.837 | 0.853 | 0.665 | 0.164 |
| Libido (BDI) | 0.452 | 0.451 | 0.510 | 0.000 |
| Social Support | 0.000 | 0.000 | 0.437 | 0.157 |
| Recent life events | 0.469 | 0.447 | 0.508 | 0.106 |
| Alcohol use (AUDIT) | 0.300 | 0.294 | 0.000 | 0.251 |

**Supplementary Table 6.** Item weights from the three ways of determining item centrality from the FGL network and factor loadings from the CFA model.

| Model | Intercept | Coefficients | | | | |
| --- | --- | --- | --- | --- | --- | --- |
|  |  | CIS-R Anxiety Sum-score | BDI-II Sum-score | Social Support Sum-score | Life Events Sum-score | AUDIT-PC Sum-score |
| Model 1 (EI 1-Step) | 2.883 | 0.300 | 0.501 | . | 0.200 | -0.072 |
| Model 2 (EI 2-Step) | 2.858 | 0.304 | 0.494 | . | 0.215 | -0.074 |
| Model 3 (PC/PR) | 7.251 | 0.324 | 0.454 | -0.642 | 0.038 | . |
| Model 4 (CFA) | 7.293 | 1.323 | 2.092 | -1.796 | 0.179 | -0.143 |
| Model 5 (ENR) | 16.641 | 1.329 | 3.867 | -0.931 | . | . |
| Model 6 (OLS) | 7.010 | 0.230 | 0.355 | -0.280 | 0.034 | -0.018 |

**Supplementary Table 7.** Coefficients and Intercepts from Models 1-6 for BDI-II score at 3-4 month outcome.

**Supplementary Table 8.** Coefficient weights and intercepts from individual item models for BDI-II score at 3-4 months.

| **Item** | **Model** | |
| --- | --- | --- |
|  | **Model 7 (ENR)** | **Model 8 (OLS)** |
| **Intercept** | 16.641 | 6.714 |
| **Anxiety (cisr)** | . | 0.015 |
| **Compulsions (cisr)** | 0.228 | 0.203 |
| **Health anxiety (cisr)** | 0.768 | 0.747 |
| **Obsessions (cisr)** | -0.077 | -0.169 |
| **Panic (cisr)** | 0.330 | 0.273 |
| **Phobia (cisr)** | 0.604 | 0.559 |
| **Somatic (cisr)** | 0.582 | 0.501 |
| **Worry (cisr)** | . | -0.061 |
| **Sadness (BDI)** | -0.033 | -0.585 |
| **Pessimism (BDI)** | 0.439 | 0.539 |
| **Failure (BDI)** | 0.412 | 0.479 |
| **Loss of pleasure (BDI)** | 0.539 | 0.856 |
| **Guilt (BDI)** | 0.555 | 0.823 |
| **Punishment (BDI)** | 0.108 | 0.071 |
| **Self dislike (BDI)** | 0.437 | 0.569 |
| **Self criticism (BDI)** | 0.086 | 0.018 |
| **Suicidal thoughts (BDI)** | 0.944 | 1.933 |
| **Crying (BDI)** | 0.383 | 0.481 |
| **Agitation (BDI)** | 0.273 | 0.582 |
| **Loss of Interest (BDI)** | 0.208 | 0.189 |
| **Indecisiveness (BDI)** | 0.336 | 0.395 |
| **Worthlessness (BDI)** | 0.265 | 0.230 |
| **Loss of energy (BDI)** | 0.788 | 1.293 |
| **Sleep (BDI)** | . | -0.096 |
| **Irritability (BDI)** | -0.278 | -0.810 |
| **Appetite (BDI)** | 0.069 | 0.075 |
| **Concentration (BDI)** | 0.316 | 0.486 |
| **Fatigue (BDI)** | 0.407 | 0.569 |
| **Libido (BDI)** | 0.222 | 0.236 |
| **Social Support** | -0.805 | -0.256 |
| **Recent life events** | 0.019 | 0.052 |
| **Alcohol use (AUDIT)** | . | 0.002 |

**Supplementary Figure 1.** Flow diagram of study selection.

**Supplementary Figure 2.** Correlation of predictions by the six models in the Test set data.


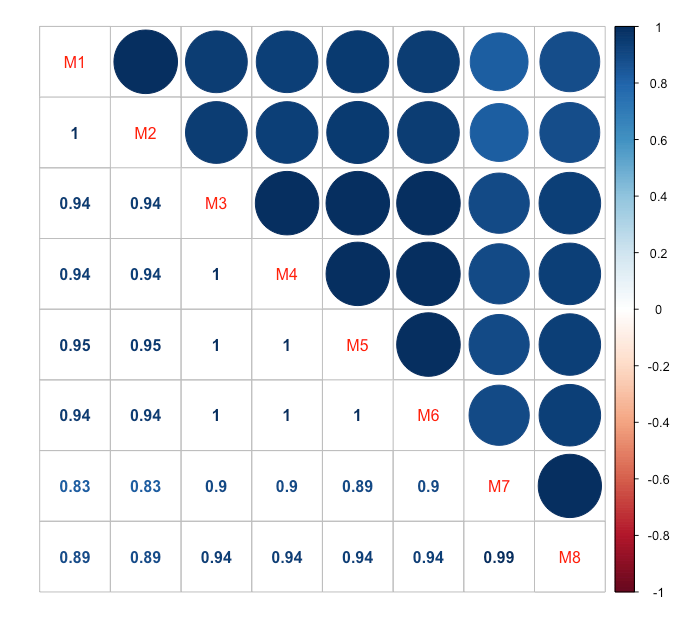


**Supplementary Figure 3.** Proportions of participants in remission at 3-4 months post-baseline in the test set (n=918) based on predicted 3-4 month BDI-II scores by each of the eight models.


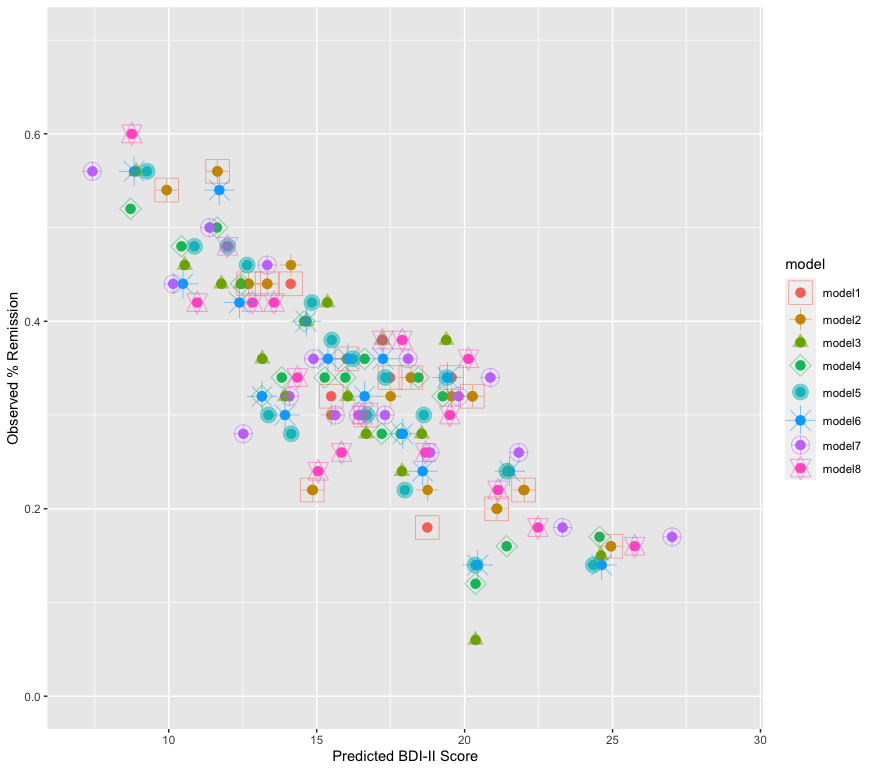

Supplement: Supplementary file 1 [file S0033291721001616sup001.docx]
